# Development of a Core Outcome Set for research studies with older adults in the Emergency Department-CREAT-ED: A multi-stage study protocol

**DOI:** 10.1186/s13063-026-09527-4

**Published:** 2026-02-16

**Authors:** K. Mc Loughlin, J. Salsberg, R. Mc Namara, E. Toomey, J. Banerjee, M. Cassarino, R. Galvin, C. O’Donnell, S. Liu, K. Dainty, M. O’Connor, D. Ryan, P. Meskell, D. Melady, K. Robinson

**Affiliations:** 1 School of Allied Health, Faculty of Education and Health Sciences, Ageing Research Centre, Health Research Institute, University of Limerick, Limerick, Ireland; 2 Health Research Board, Trials Methodology Research Network (HRB-TMRN), Limerick, Ireland; 3 School of Medicine & Health Research Institute, University of Limerick, Limerick, Ireland; 4 Emergency Department, St. Vincent’s University Hospital, Dublin, Ireland; 5 School of Medicine, University College Dublin, Dublin, Ireland; 6 Centre for Health Research Methodology, School of Nursing and Midwifery, University of Galway, Galway, Ireland; 7 Emergency Department, University Hospitals of Leicester NHS Trust, Leicester, UK; 8 Department of Public Health Sciences, University of Leicester, Leicester, UK; 9 School of Applied Psychology, University College Cork, Cork, Ireland; 10 School of Medicine, Faculty of Education and Health Sciences, University of Limerick, Limerick, Ireland; 11 Department of Emergency Medicine, Massachusetts General Hospital, Boston, USA; 12 Harvard Medical School, Boston, USA; 13 North York General Hospital, Toronto, Canada; 14 Institute of Health Policy, Management and Evaluation, University of Toronto, Toronto, Canada; 15 Emergency Department, Limerick EM Education Research Training (ALERT), University Hospital Limerick, Limerick, Ireland; 16 Department of Family and Community Medicine, Schwartz Reisman Emergency Medicine Institute, Toronto, ON Canada

**Keywords:** Emergency medicine, Older adults, Core Outcome Set, Outcome Assessment (Health Care), Real-Time Delphi

## Abstract

**Background:**

Older adults represent a growing proportion of Emergency Department (ED) attendees and experience high rates of adverse outcomes post discharge from the ED such as functional decline, increased risk of institutionalisation and unplanned hospital readmissions. In response to these issues, there has been a proliferation of studies evaluating various interventions to mitigate these adverse outcomes. However, this expansion of research has been hampered by a lack of agreement on ‘what to measure’ in these studies and a lack of attention to the measurement of patient/ service user reported outcomes. To address these issues, we aim to develop a Core Outcome Set (COS) for intervention studies based, or initiated, in the ED with older adults.

**Methods:**

A five-stage approach will be undertaken to develop the COS in conjunction with a Public and Patient Involvement (PPI) panel of older adults and family carers. To generate a long list of outcomes, three stages will be undertaken.

1. A scoping review to identify commonly reported outcomes in intervention studies based/initiated in the ED, associated instruments and measurement timepoints.

2. A rapid qualitative evidence synthesis to identify outcomes of importance to older adults attending the ED.

3. Participatory research methods will be employed to identify outcomes of relevance to older adults and their families/ caregivers, health care Providers and research stakeholders.

To achieve consensus on outcomes and associated instruments to include in the COS, a further two stages will be undertaken.

4. A Real-Time Delphi survey study and consensus meeting with key stakeholders to reach consensus on inclusion of outcomes in the COS.

5. A search for existing instruments to evaluate each outcome identified in stage 4 will be conducted. Identified instruments will be evaluated, and a consensus meeting will be held to select one outcome measure instrument for each outcome included in the COS.

**Discussion:**

A COS for research with older adults in the ED will serve to enhance future clinical trials, systematic reviews and clinical guidelines by enhancing the availability of comparable data and by ensuring outcomes that matter to all stakeholders are represented.

**Trial registration:**

Development of a Core Outcome Set for research studies with older adults in the Emergency Department-CREAT-ED (COMET) initiative 2749 COMET Initiative | Development of a Core Outcome Set for research studies with older adults in the Emergency Department-CREAT-ED. https://www.comet-initiative.org/Studies/Details/2749.

## Background

In 2015, the World Health Organisation (WHO) produced the first world report on ageing and health highlighting that people are living longer worldwide, in particular those in high income countries [1]. In Ireland, it is predicted that we will see a 59% population growth in those aged 65 and older between 2016 and 2031 [2].

In line with these changing demographics, the number of Emergency Department (ED) visits by older adults is increasing. This cohort accounts for up to one quarter of all ED attendees [3]. The prevalence of frailty among older adult ED attendees has been reported to be between 40% and 58.5% [4, 5]. Older adults with frailty often present to the ED with non-specific complaints including falls, general weakness/fatigue and delirium [6]. Of concern, older adults experience high rates of adverse outcomes post ED discharge, including unscheduled return ED visits, functional decline, nursing home admission and death [7].

While ED visits are associated with adverse outcomes for older adults [7, 8], an ED visit also presents an opportunity to deliver interventions or enact care pathways. For example, in recent years, several studies have explored the effectiveness of various discharge planning interventions in the ED. In their review of ED interventions for older adults, Hughes et al. [9] define discharge planning as a process that occurs entirely within the ED that is time-sensitive and focuses on developing and formalising a care plan before the older adult leaves the ED. Discharge planning may include elements such as geriatric consultation or assessment, the provision of educational material for the older adults and/or their caregiver, and a follow-up plan [9].

Research on this type of ED-based or ED-initiated intervention with older adults is proliferating, as demonstrated by the growing number of systematic reviews examining interventions for older adults in the ED [3, 9, 10]. Despite this proliferation, there is a lack of agreement on ‘what to measure’ and ‘how to measure’ outcomes in studies with this population in the ED. For example, a systematic review of the impact of mobility assessments of older adult ED patients was unable to undertake a meta-analysis due to heterogeneity in study populations and outcomes [11]. Another systematic review of the impact of early assessment and intervention by teams involving health and social care professionals (HSCPs) working in the ED with older adults found no studies investigating ED length of stay or cost-effectiveness, and inconsistency around measurement of several outcomes such as incidence of hospital admission, length of stay, ED re-presentation rate, community referrals, mortality, patient and staff satisfaction and health related quality of life [12]. A more recent umbrella review which included nine systematic review papers focused on interventions to reduced adverse outcome in older adults following ED discharge, also found heterogeneity in outcome domains and tools utilised to measure reported outcomes [10]. This is concerning as in the field of Geriatric Emergency Medicine, there is a lack of consensus to guide practitioners [13, 14].

Reviews have also noted the heterogeneity of timing of follow up assessment in trials with older adults in the ED. For example, ED return visit was an outcome in seven of nine reviews in the aforementioned umbrella review measured from one month post visit to 18 months [10].

Lack of attention to patient reported outcomes (PRO) in studies with older adults in the ED has also been noted [3, 10]. A PRO can be defined as;* ‘any report of the status of a patient’s health condition that comes directly from the patient, without interpretation of the patient’s response by a clinician or anyone else’* [15]. The umbrella review referenced above reported that only four of the nine reviews reported patient experience or satisfaction, with significant heterogeneity in the tools used [10].

Previous qualitative research using participatory methods has highlighted that older adults and healthcare providers have different priorities related to care in the ED [12]. Older adults valued positive communication and relational aspects of care, in contrast healthcare providers focused more on technical aspects of care and process outcomes [12]. A further study focused on health-related quality of life outcomes valued by older adults following an ED visit emphasised their priorities as returning to functional baseline and the importance of communicating diagnosis, prognosis and signposting for follow up as required [16]. Similarly, a qualitative study of the goals of older adults with frailty and their caregivers when they access urgent care found they wanted their medical problem diagnosed and resolved, information and tests and treatment, a well-planned discharge home and that the retain mobility, function and normal activities [17]. Thus, it is imperative that outcomes valued by older adults are also included in intervention studies with this population in the ED setting.

The outcome heterogeneity discussed above has implications for reviewing research evidence and for generating policy implications. The systematic reviews cited above were limited in their ability to synthesise outcomes and generate clear recommendations for practice due to outcome heterogeneity. At the conclusion of their systematic review, Hughes et al. [9] note that *‘careful selection of measures that are both appropriate for a heterogeneous population and responsive to change is critical. Developing common measures to be used across studies may also help advance the field, as has been done within other geriatric research areas’* ([9] p.1524).

The development of a Core Outcome Set (COS) is an established methodology to address issues with outcome heterogeneity in research. A COS can be defined as “*an agreed standardised set of outcomes that should be measured and reported, as a minimum, in all clinical trials in specific areas of health or health care*” [18]. A well-developed COS can help reduce research waste by improving the comparability of study findings and can protect against the selective reporting of outcomes or outcome reporting bias [19]. Perhaps the greatest strength of a COS is its ability to ensure the outcomes measured in trials align with what matters most to service users and patients. This can only be achieved through a collaborative COS development process that meaningfully involves key stakeholders, including healthcare providers, researchers, policymakers, research/service funders and patients/service users.

The primary aim of this study is to develop an evidence and consensus-based Core Outcome Set (COS) for research studies with older adults in the ED.

## Methods

### Protocol and prospective registration

This six-stage study protocol was prospectively registered with the COMET Initiative on July 11th, 2023, and is available at COMET Initiative | Development of a Core Outcome Set for research studies with older adults in the Emergency Department-CREAT-ED (registration number 2749) (comet-initiative.org). This protocol was developed and reported in adherence with the Core Outcome Set-STAndardised Protocol (COS-STAP) Items checklist [20] (Appendix). The design and conduct of this multi-stage study will align with the Core Outcome Set-STAndards for Development (COS-STAD) [21], a set of eleven minimum standards to be followed by COS developers and the more detailed guidance provided by the COMET (Core Outcome Measures in Effectiveness Trials) handbook [18]. The developed COS will be reported according to the Core Outcome Set-STAndards for Reporting (COS-STAR) guidelines [22].

### COS scope

The research or practice setting(s) in which the COS is to be applied is research on interventions delivered in, or initiated in, the ED in high-income countries. The health condition(s) covered by the COS are inclusive of any presenting complaint in the ED setting including non-specific or undifferentiated complaints in older adults. Health conditions not covered by the COS include conditions where there is a well-defined and established treatment or care pathway within the ED for their presenting complaint, such as stroke, myocardial infarction (M.I.) and trauma.

The population(s) covered by the COS are older adults aged 65 years or older. The COS will also cover studies where the recruited sample are healthcare providers delivering an intervention to older adults in the ED where outcomes related to older adults are examined. The COS will not cover studies where the recruited sample are healthcare providers delivering an intervention to older adults in the ED and outcomes only relevant to healthcare providers (e.g. healthcare provider stress) are examined.

The intervention(s) covered by the COS includes an intervention (any care, model of care, or management strategy) commenced or completed with older adults in the ED setting or focused on health care professionals with the aim to improve outcomes related to older adults in the ED setting (also known as Accident and Emergency (A&E), casualty and the Emergency Room). Studies of surgical interventions or studies exploring the introduction of new drug therapies or studies of emergency medical intervention will be excluded. Studies where the primary focus is to validate or implement a screening tool (such as those used to identify frailty or delirium), where an intervention to address the issue screened for is not implemented with older adults will be excluded.

### Public and patient involvement (PPI)

PPI was central to study conceptualization. The overall study proposal was informed by previous participatory research conducted with older adults who had used ED services at two clinical sites in Ireland which highlighted deficiencies in relational care including deficiencies in healthcare provider communication in the ED [23] and ED experiences of University of Limerick Ageing Research Centre PPI panel members. Three older adult/family carer co-researchers from the Ageing Research Centre PPI panel participated in study design meetings which led to the grant application for this research and will be involved as co-researchers and co-authors in every phase of the study. The three older adult/family carer co-researchers also sit on the project steering committee.

### Design

The research aim will be addressed through 5 sequential stages:

#### Stage 1: Scoping review

##### Objective

Stage one aims to identify outcomes and outcome measurement instruments reported in experimental studies with older adults in the ED. This will allow us to compile an initial long list or pool of potential outcomes to consider for inclusion in a core outcome set. To address this a scoping review will be conducted in accordance with the Joanna Briggs Institute guidelines [24]. A protocol for this scoping review is available at OSF | Outcomes and outcome measure instruments reported in studies with older adults in the Emergency Department: Protocol for a scoping review https://osf.io/q68c7/overview. 

##### Search strategy

An international trial register (ClinicalTrials.gov) and three electronic databases (MEDLINE, CENTRAL and CINHAL) will be searched from 2003 onwards using keywords related to 2 core concepts (older adults and the emergency department). We decided to focus on studies published in the last 20 years for pragmatic reasons as we are aware from recent reviews that a large body of experimental research on this topic exists [9, 10] and we want the long list of outcomes generated at this stage to reflect contemporary research.

##### Study selection

Two members of the research team (KMcL, KR) will conduct searches, screening and data extraction independently. Any disagreements by the reviewers will be resolved through discussion, and if unable to achieve consensus, an additional reviewer will be consulted (RG). If any study includes missing or incomplete reports, an attempt will be made to contact the author.

##### Eligibility criteria

This scoping review will include studies that recruit populations as per the scope of the overall COS reported above in section ‘COS scope’. Longitudinal experimental (randomised, nonrandomised, quasi-experimental and pre/post studies) study designs will be included. This review will include studies of interventions in the ED as per the scope of the overall COS reported above in section ‘COS scope’.

##### Data extraction

Data on study characteristics and outcomes/outcome domains assessed, outcome measurement instruments and timing of outcome measurement will be extracted using a custom developed template.

An outcome is defined as ‘a measurement or observation used to capture and assess the effect of treatment such as assessment of side effects (risk) or effectiveness (benefits)’ [18]. An outcome refers to ‘what is being measured’. It is also referred to as a construct or domain. In the context of a clinical trial, it refers to what is being measured on trial participants to examine the effect of exposure to a health intervention [25]. Examples of commonly measured outcomes or outcome domains in studies with older adults in the ED include mobility, function and quality of life. Examples of outcome measurement instruments include the Barthel Index [26] and the EQ-5D [27].

##### Data analysis

A PRISMA-ScR flow chart [28] will be populated to report the selection process. Extracted data will be presented in tables and summarised narratively. The frequency of reporting of outcomes, measures and assessment timepoints will be numerically summarised.

A long list of outcomes, their definitions and measurement instruments will be generated from this scoping review.

#### Stage 2: Rapid qualitative evidence synthesis

A rapid Qualitative Evidence Synthesis (rQES) will be completed to summarise research findings related to older adults’ ED care priorities, or their experiences related to or following an ED visit to identify outcomes that are valued by older adults. This stage will complement stage 1 (scoping review) by potentially contributing additional outcomes of importance to older adults not reported in studies to date. Guidance on the conduct of rapid QES by Booth et al. [29] will be followed in the conduct of this review. A rapid QES was selected given the timeline of the broader COS development project. A rapid QES will allow us to obtain timely findings that can be incorporated in a more expedited manner compared to a full review process which typically takes 6 months to 2 years to complete [30]. Rapid QES also aligns with our aim, which is descriptive rather than interpretative. In comparison to a full QES our rapid review will be expediated using a limited number of databases for searches, and the use of a single reviewer for search result screening and data extraction.

##### Search strategy

A systematic electronic database search will be performed in Scopus plus CINAHL plus ProQuest Dissertations and Theses Global (three-database combination); this approach has been found to retrieve 92% of relevant studies [31]. Databases will be searched from the year 2000 to present day using keywords and qualitative research methodological filters in line with rapid QES methodology. Included studies in two recent evidence syntheses on related topics will also be reviewed (a synthesis of qualitative studies on older adults’ experiences of transition home from the ED [32] and a synthesis of published studies of expectations and preferred outcomes from emergency care among older people or their caregivers [33]. An information specialist will peer review one database search strategy. Reference lists of included papers will also be reviewed.

##### Inclusion criteria

English language peer-reviewed papers reporting primary qualitative research on older adults’ ED care priorities or their experiences related to or following an ED visit will be included. The limitation of only including English papers only will be acknowledged in the study limitations. Studies of mixed aged participants will be included where the experiences of older adults can be separately extracted.

##### Study selection

One member of the research team will independently assess the titles, abstracts and full texts identified by the search using Rayaan software. A proportion of results (10%) will be assessed by a second reviewer. The selection process will be recorded in sufficient detail to complete a PRISMA flow diagram [34].

##### Data extraction

One researcher will independently extract data using an extraction form designed for the synthesis. Data will be extracted from a proportion of included studies (10%) by a second reviewer.

##### Quality appraisal

Included papers will be assessed for methodological quality by two researchers independently (KMcL, KR) using the Critical Skills Appraisal Program (CASP) tool for qualitative studies [35].

##### Analysis

A ‘best-fit framework approach’ will be used to analyse and synthesise the evidence. This approach is one recommended approach to analysis in rapid QES [29]. Findings from the included studies will be uploaded to NVivo 11 Pro (QSR International Pty Ltd). The five stages of the best-fit framework are as follows: familiarisation, identifying a thematic framework, indexing, charting, mapping and interpretation will be followed. After familiarisation with the included studies, the first author will develop upfront categories focused on outcomes identified in the scoping review (Stage 1) and findings of previous relevant reviews in this area [32, 36, 37]. Outcomes reported by older adults in these papers will be combined with outcomes identified in stage 1 and grouped into categories. An older adult co-researcher will work with the lead author to refine this a priori framework. This framework will be further refined as analysis of the included papers progresses. The Grading of Recommendations, Assessment, Development and Evaluations-Confidence in the Evidence from Reviews of Qualitative research (GRADE-CERQual) approach will be used to assess confidence in the findings [38]. Outcome domains identified in stage 2 will be added to the list generated in the scoping review (stage 1).

#### Stage 3: Qualitative, participatory study

The third stage will focus on identifying outcomes valued by key stakeholders (older adults, their families/caregivers, researchers and health care providers) when evaluating the care and experiences of older adults in the ED context. To our knowledge, only one qualitative study to date focuses exclusively on older adults’ views on important outcomes following ED attendance [39]. Previous qualitative research has revealed challenges researchers face in selecting outcome and outcome measurement instruments in trials [40]; however, no study to our knowledge has explored this in the context of ED research with older adults. In this stage, the perspectives of stakeholder groups who may not be represented in the available qualitative evidence will be elicited, particularly, healthcare providers and academic researchers.

##### Research design

A qualitative, participatory design will be employed adhering to the Consolidated Criteria for Reporting Qualitative Research (COREQ) guidelines [41]. A form of focus group, a World Café, will be conducted with mixed groups of stakeholders at two locations in Ireland.

The World Café; a group discussion format, is designed to be as inclusionary as possible and facilitate mutual dialogue and learning among a relatively large group of participants [42]. Individual interviews will also be offered to potential participants who cannot attend scheduled World Cafe’s.

Participants will include older adults and family members/carers of older adults who have attended the ED in Ireland in the previous 3 years either as patient or relative and healthcare providers including ED doctors and nurses, HSCPs working in the ED or in the hospital and pre-hospital staff (advanced paramedics or emergency medical technicians) in full- or part-time employment in the hospital for at least 12 months (thus, familiar with the ED/hospital procedures).

We will recruit participants through purposive sampling at two ED sites in Ireland until data saturation; the point where new data no longer contributes to the analysis [43, 44] is agreed by two researchers. Data analysis will commence in tandem with data collection.

The World Café discussion prompts will be designed to foster dialogue on outcomes that these diverse stakeholders consider important when evaluating the care and experiences of older adults in the ED context.

##### Data analysis

Participants’ responses in the group (World Cafe) and individual interviews will be audio-recorded electronically and transcribed in full. This data will be entered into the qualitative data analysis software NVivo 11 Pro (QSR International Pty Ltd). A descriptive inductive approach to data analysis will be employed to provide a rich and detailed account of the data, adhering to a six-stage guide to reflexive thematic analysis guided by the research aim [45]. Older adult co-researchers will be involved in data analysis working alongside the lead author in the later stages of analysis. Participants will be recruited via gatekeepers at both ED sites.

All outcome domains identified through this analysis will be added to the long list generated in earlier stages.

#### Stage 4: Real-Time Delphi study

The primary goal of this stage is to achieve consensus among stakeholders on the most important outcomes to include in the COS using the Real-Time Delphi method. International key stakeholders including policy makers/health service funders, patients, family carers, HCPs and community stakeholders will participate in a Real-Time Delphi study and consensus meeting to achieve consensus on inclusion of outcomes generated in stages 1–3 in the COS. All outcomes identified in stages 1–3 will be included in the Real-Time Delphi.

Multiple rounds of Delphi surveys are commonly used in COS development to allow participants rate the importance, of various outcomes. Ratings given in the previous round are presented in subsequent rounds to allows participants to consider others’ opinions before having the option to re-rate the outcome or retain their original rating. A trial comparing multi-round Delphi with a Real-Time Delphi in COS development indicated considerable benefits of the Real-Time Delphi approach [46]; thus, we are adopting this approach.

### Developing the survey

We will review all identified outcomes (from stages 1–3), and consider along with older adult co-researchers, if there is a meaningful categorisation. Collaboratively, we will develop lay-language descriptions that will appear alongside the identified outcomes where necessary in the survey. The survey will be piloted on two stakeholders from each group through face-to-face interviews to obtain input on categorisation of survey items and the adequacy of lay-language summaries.

It is likely there will be a large number of potentially relevant outcomes to rate which may be burdensome for older adult participants in the Real-Time Delphi. Based on the reflections of another COS development team who worked with older adult participants, we will reduce the number of outcomes presented during the Delphi stage through the use of multi-stakeholder workshops with a smaller number of participants to review outcomes ahead of the Delphi [47].

### Sampling/recruitment

Participants will be identified and recruited through various means: (1) Older adult ED service users and their partners or family members will be recruited through patient-advocacy groups, open advertisement in community locations, social media advertisement and through advertisement at clinical locations. Additionally, older adult and caregiver participants recruited during stage 3 will be invited to participate in the survey; (2) researchers/trialists will be recruited through author lists of papers included in stages 1 and 2; and (3) healthcare providers will be recruited through members of national and international societies of Emergency Medicine and participant lists published by international conferences, through social media and through known contacts of the research group.

To maximize the validity of our findings, adequate representation from each stakeholder groups will be sought. However, there are currently no definitive recommendations about the ideal group size for a Delphi survey with aim if achieving consensus on a COS Therefore, we will aim to recruit a minimum of 25 individuals from each stakeholder group to ensure diverse representation in line with recommendations from the COMET Handbook. However, the emphasis will be on recruiting as many as possible of each stakeholder groups across global locations.

### Data collection

The survey will be built, administered and distributed to Delphi panel members using Calibrum software.

Recruitment will be monitored throughout the survey period to assess both overall response rates and the proportion of respondents who are older adults and/or family caregivers. If the predefined minimum target of 25 older adults/family caregivers is not achieved, or if this group represents less than 15% of the total respondent pool compared with researchers and healthcare providers, this will be taken as evidence of under-representation. In such cases, active and targeted recruitment strategies will be implemented, including offering participation via telephone or face-to-face Delphi surveys in familiar and accessible settings such as local community venues to support engagement [47]. These alternative participation options will be clearly communicated in all recruitment and advertising materials for the Real-Time Delphi survey.

### Real-Time Delphi

The design of this stage is based on the published protocol [48]. Before starting the Real-Time Delphi survey, a group of stakeholders (at least five from each of the three stakeholder groups) will complete the initially developed survey. This provides consensus information that can be shared with subsequent RT Delphi participants.

Participants will score each outcome on a Likert scale to indicate its importance, and labelled as ‘not important’, ‘important but not critical’ and ‘critical’ [49]. Participants will also be asked to identify which stakeholder group they best identify with and provide demographic details. One open question will ask for suggestions of any other outcome for inclusion. These suggestions will be reviewed by the steering group within 2 days and added to the Real-Time Delphi if proposed by two or more participants.

Following their rating of each outcome, participants will be presented with real-time feedback. This will include their personal rating, the overall group’s rating and the rating for each stakeholder group. Participants will then have the option to either maintain their initial rating or modify it based on the real-time feedback. An option to provide a rationale on the selected rating if the participant wishes will also be included [47]. The survey will remain open for a fourteen-week period during which time participants can revisit the portal, view other participants responses in real time and re-rank their responses if they wish. Participants will be reminded by email to revisit the survey twice during the survey period.

### Analysis

Responses will be analysed and organised by stakeholder group; descriptive and frequency statistics will be calculated for each scale item within each stakeholder group.

### Defining and assessing consensus

A clear definition of what constitutes consensus is required a priori to reduce potential bias in the interpretation of the results in keeping with the COMET handbook guidelines [18]. These guidelines were utilised in assessing the degree of consensus required for inclusion in this COS.

After the Real-Time Delphi for each stakeholder group, we will assign each outcome to one of three categories, as described in the COMET handbook [18]:

1. Consensus to include the following: where >/= 70% participants in a stakeholder group rate the outcome as critically important and </= 15% or fewer rate the outcome as limited importance.
2. Consensus to exclude the following: >/= 70% participants in a stakeholder group rate the outcome as limited importance and </= 15% rate the outcome as critically important.
3. No consensus.

#### Stage 5 Online consensus meeting

The results of the Delphi survey will be discussed at an online consensus meeting with an invited sub-group of participants from all stakeholder groups. The project steering committee will ensure that number of attendees from each stakeholder group are balanced to support equitable participation. This same group will also be invited to a further consensus meeting in the next stage [6] to agree specific instruments to measure each domain.

In addition to those groups who completed the survey at this stage, other stakeholders including health/research funders and policymakers will be invited to the consensus meeting.

The online meeting will begin with introductions and explanation of the process. The meeting will be facilitated by two researchers who will not participate in voting. All outcomes that achieved consensus and failed to achieve consensus in stage 4 will be discussed, and voting will be used to agree the final COS. In line with the process used in the development of other COS, consensus will be described as where at least 70% of participants vote for the outcome to be including with at least one PPI representative in agreement [50]. Further meeting(s) will be scheduled if consensus is not achieved.

### Ethics

Ethical approval for stages 3 to 5 has been granted from two Hospital/Heath Service Executive Research Ethics Committees and the Faculty of Education and Health Science Research Ethics Committee at the University of Limerick (REC reference 24/59; REC reference 0094; REC reference 2024_09_18_EHS (FA)) on 25.10.24; 6.11.24 and 11.10.24, respectively.

#### Stage 6 Selection of outcome measure instruments

To identify appropriate outcome measurement instruments for the outcomes included in the COS, we will follow the rigorous guidelines developed through a joint initiative between the COnsensus-based Standards for the selection of health Measurement INstruments (COSMIN) initiative and the Core Outcome Measures in Effectiveness Trials (COMET) initiative [51] for the selection of such instruments. An outcome measurement instrument refers to ‘how the outcome is being measured. It is a tool to measure a quality or quantity of the outcome. The tool can be a single question, a questionnaire, a score obtained through physical examination, a laboratory measurement, a score obtained through observation of an image, etcetera’ [51].

The process will involve four steps: (1) definition of the outcome construct by the project steering committee, (2) finding existing outcome measurement instruments through literature searches and/or published reviews of outcome measurement instruments, (3) quality assessment of available outcome measurement instruments by means of the evaluation of the measurement properties and feasibility aspects of the instruments, (4) selection of one outcome measurement instrument per outcome domain. Step 4 will be conducted via an online consensus meeting with the same participants as attended the online consensus meeting in stage 5.

### Dissemination and implementation strategy

Abstracts will be prepared for Irish and international conferences. To disseminate the COS to researchers internationally a manuscript will be submitted to a relevant high quality, open access journal such as Age and Aging, and an infographic will be developed to disseminate the COS via social media. The PPI contributors will co-author or be acknowledged in the final publication in line with guidance on authorship with patient partners [52]. The PPI contributors for this study have extensive experience, having contributed to multiple studies on emergency department care for older adults over the past five years. To support those who wish to co-author, we will provide clear information about the publication and peer-review process, co-develop terms of reference outlining roles and expectations, discuss authorship early and offer flexible communication and timelines to accommodate individual preferences, capacities and resources. This protocol has been registered with COMET, and the final COS will be published on their website. To share findings with older adults and their families, a lay summary of key findings will be prepared for dissemination jointly by the first author in collaboration with PPI collaborators and disseminated to all stakeholders who participated in the COS development process and to relevant organisations of/for older adults such as the International Federation on Ageing and Aging. A policy brief will be developed and distributed to the Director of National Clinical Strategy & Programmes and relevant National Clinical Programmes (Older Persons and Emergency Medicine) in Ireland and sent to international bodies including the European Geriatric Medicine Society (EuGMS), the European Society for Emergency Medicine (EUSEM) and the International Federation for Emergency Medicine (IFEM). Co-authors of this protocol include international experts in the area of Geriatric Emergency Medicine and will also disseminate the COS worldwide through their networks.

### Expected timeframe

At protocol submission (December 2024), the scoping review was in progress and pilot searches for the QES had been completed. The rapid QES will run for six months and is scheduled for completion in Quarter 2, 2025. Recruitment for the qualitative participatory study will begin in Quarter 1, 2025, with completion expected in early Quarter 3, 2025. These first three stages will overlap but will all conclude before development of the Real-Time Delphi survey. A three-month period following Phase 3 will be used to refine the long list of outcomes and develop and pilot the Delphi survey, which will launch in Quarter 4, 2025, and remain open until the end of Quarter 1, 2026. Stage 5 consensus meetings will take place at the start of Quarter 3, 2026, and selection of measurement instruments will be completed by the end of Quarter 4, 2026. Dissemination activities are planned for Quarter 1–2, 2027.

## Discussion

This protocol describes the methodology for developing a Core Outcome Set (COS) for research studies involving older adults in the ED. Despite the growing body of research in this area, there is currently no consensus on the core outcomes that should be measured in studies with older ED patients, from the perspectives of all important stakeholders. The potential impact of this study is significant. Core Outcome Sets are rapidly becoming an established element of the infrastructure of intervention and clinical trial research. Completed rigorously, and with meaningful involvement of all stakeholders, COS have the potential to focus research on the outcomes that matter most to key stakeholders.

Several related core outcome sets (COS) exist for specific interventions in older adults, such as deprescribing [53] and nutritional interventions for those with/at risk of malnutrition [54]. For studies evaluating these interventions in the ED setting, these existing COS could be used alongside the COS developed in this study. Likewise, for trials focused on specific conditions presenting in the ED, relevant condition-specific COS—for example, the COS for Dementia with Lewy Bodies [55] or the COS for frailty [56]—could be combined with our COS to ensure comprehensive outcome selection.

Once published, a COS for research with older adults in the ED has the potential to enhance the conduct and reporting of future clinical trials, systematic reviews and clinical guidelines by enhancing the availability of comparable data and by ensuring outcomes that matter to all stakeholders are represented.

### Strengths and limitations

As with all research, it is important to acknowledge potential strengths and limitations to this proposed work.

Throughout every stage of the development of this COS, there is active PPI involvement including multiple PPI members on the stakeholder group. An additional key strength of this COS development project is the composition of the interdisciplinary and international steering group/authorship group.

Although the aim is for this COS to be applied internationally, we acknowledge the limitations in limiting its focus to high income countries and English language respondents. Efforts will be made to involve stakeholders in multiple countries and continents, but this may not always be possible. This may also be further limited by World Café conducted in Ireland only but again this is reflective of the scope of the resources available.

### Trial status

Protocol version 2.0 November 2025.

The scoping review is complete and the rQES will commence in the coming months. Recruitment of participants for stages 3–5 is expected to commence in January 2025.

### Administrative information

This research is funded through the Health Research Board of Ireland of Ireland (HRB) Investigator Led project award (ILP-HSR-2022–010). The funders had no role in study design, data collection and analysis, decision to publish or preparation of the manuscript.

## Data Availability

The final core outcome set will be submitted for peer review and reported according to the Core Outcome Set-Standards for Reporting (COS-STAR).

## Appendix 1

Core Outcome Set-STandards Protocol Items: The COS-STAP Statement Checklist

**Table 1:**

Section/topic	Item No.	Checklist item	Reported on page number
Title/abstract			
Title	1a	Identify in the title that the paper describes the protocol for the planned development of a COS	1
Abstract	1b	Provide a structured abstract	1-2
Introduction			
Background and objectives	2a	Describe the background and explain the rationale for developing the COS, and identify the reasons why a COS is needed and the potential barriers to its implementation	2-3
	2b	Describe the specific objectives with reference to developing a COS	3
Scope	3a	Describe the health condition(s) and population(s) that will be covered by the COS	3
	3b	Describe the intervention(s) that will be covered by the COS	3
	3c	Describe the context of use for which the COS is to be applied	3
Methods			
Stakeholders	4	Describe the stakeholder groups to be involved in the COS development process, the nature of and rationale for their involvement and also how the individuals will be identified; this should cover involvement both as members of the research team and as participants in the study	3,5-7
Information sources	5a	Describe the information sources that will be used to identify the list of outcomes. Outline the methods or reference other protocols/papers	4-6
	5b	Describe how outcomes may be dropped/combined, with reasons	6-7
Consensus process	6	Describe the plans for how the consensus process will be undertaken	6-7
Consensus definition	7a	Describe the consensus definition	7
	7b	Describe the procedure for determining how outcomes will be added/combined/dropped from consideration during the consensus process	6-7
Analysis			
Outcome scoring/feedback	8	Describe how outcomes will be scored and summarised, describe how participants will receive feedback during the consensus process	7
Missing data	9	Describe how missing data will be handled during the consensus process	7
Ethics and dissemination			
Ethics approval/informed consent	10	Describe any plans for obtaining research ethics committee/institutional review board approval in relation to the consensus process and describe how informed consent will be obtained (if relevant)	7-8
Dissemination	11	Describe any plans to communicate the results to study participants and COS users, inclusive of methods and timing of dissemination	8
Administrative information			
Funders	12	Describe sources of funding, role of funders	10
Conflicts of interest	13	Describe any potential con	9

## Abbreviations

COMET: Core Outcome Measures in Effectiveness Trials
COS: Core Outcome Set / Core Outcome Sets
COS-STAP: Core Outcome Set-Standardised Protocol Items
COS-STAD: Core Outcome Set-Standards for Development
COS-STAR: Core Outcome Set-Standards for Reporting
CASP: Critical Skills Appraisal Program tool
ED: Emergency Department
GRADE-CERQual: The Grading of Recommendations, Assessment, Development and Evaluations-Confidence in the Evidence from Reviews of Qualitative research
HSCPs: Health and Social Care Professionals
HCP: Health Care Professional
HRB-TMRN: Health Research Board Trials Methodology Research Network
PPI: Public and Patient Involvement
PRO: Patient Reported Outcomes
rQES: Rapid Qualitative Evidence Synthesis
